# Factors Affecting Quality of Work Life in a Sample of Cancer Survivor Female Nurses

**DOI:** 10.3390/medicina56120721

**Published:** 2020-12-21

**Authors:** Ju Hyun Jin, Eun Ju Lee

**Affiliations:** 1 Research Institute of Nursing Science, Keimyung University, Daegu 42601, Korea; dominicajin@hanmail.net; 2 College of Nursing, Keimyung University, Daegu 42601, Korea

**Keywords:** cancer survivors, fatigue, job stress, workplace spirituality, quality of work life

## Abstract

*Background and objectives:* Identifying the factors affecting the Quality of Work Life (QWL) of cancer survivor female nurses is important and necessary to overcome the various challenges experienced by these professionals upon returning to work following recovery from the disease. Therefore, this study aimed to identify the factors affecting the level of nurses’ QWL. *Materials and Methods:* A cross-sectional survey was conducted among 115 registered female nurses who had survived cancer, in general hospitals and clinics in South Korea. SPSS statistics version 21 was used for ordinary least squares, and Stata version 12.0 was used for quantile regression analysis. *Results:* Workplace spirituality affected all quantiles of QWL except the 90% quantile; fatigue was an affecting factor in the 20%, 30%, and 70% quantiles; and job stress in the 20%, 30%, 40%, and 60%, 70%, 80% quantiles. For workplace spirituality, the effect size was 0.33 (*p* < 0.001) in the 10% quantile, increasing to 0.45 (*p* < 0.001) in the 80% quantile. *Conclusions:* Based on the results of this study, suggestions for clinical practice include providing the mediating strategies and programs to manage fatigue and job stress as well as workplace spirituality. Job-related factors such as shift work should also be considered.

## 1. Introduction

In 2017, according to the South Korea National Cancer Information Center, the leading types of cancer among men were gastric cancer, lung cancer, and colorectal cancer, and those among women were breast cancer, thyroid cancer, and gynecologic cancer [1]. Female cancers are consistently on the rise, due to factors such as irregular lifestyle, changes in diet, increased stress among people in modern society, decreased breastfeeding, and changes in sexual cultures [2]. Traditionally, in South Korea, the nursing profession is predominated by women, and female nurses are vulnerable to various health problems due to irregular lifestyles resulting from long work shifts, and fatigue and stress from excessive workload [3]. According to a survey conducted by the Korean Nurses Association, nurses seem to experience health problems such as cancer, heart disease, digestive disorders, urinary infections, and vaginal infections [3,4]. Furthermore, prior research has indicated that nurses who worked rotating shifts after midnight had a significantly increased risk for breast cancer, compared to nurses who worked only day shifts [5]. Based on these findings, nurses have a higher incidence of chronic diseases such as cancer, compared to other professionals, and this is particularly evident in female nurses [3,4,5].

Although there is no mass-scale statistical data on cancer incidence among Korean nurses, in 2017 the incidence of cancer in women was 428.6 per 100,000 population with a consistently rising 5-year survival rate at 70% in South Korea [1]. While half of these cancer patients are work-capable individuals, the rate of their return to work is merely 30.5%, which is markedly lower than the rate of 63.5% reported in other countries [6]. Further, even those who do return to work are faced by an array of challenges, and for this reason, the unemployment rate among cancer survivors is a whopping 43% [7].

Quality of work life (QWL) refers to an individual’s subjective satisfaction experienced in work life [8]. Improving QWL can increase the likelihood of cancer survivors’ returning to work, improve their job engagement, and reduce turnover rate [9]. However, even after returning to work, cancer patients experience physical functional limitations and reduced cognitive functioning due to cancer treatment, and the financial and temporal burden of managing work and treatment schedules causes fatigue and intensifies job stress [10]. Such fatigue and job stress may, in turn, further deteriorate QWL. While fatigue is a routine outcome following physical and mental exhaustion among normal workers [11], it is the most common symptom experienced by nurse cancer survivors upon returning to work [9,12]. Indeed, fatigue is an important factor involved in returning to work, turnover intention, and resignation from work among cancer survivors, and thus it is the most crucial side effect of cancer treatment that must be managed promptly [9,13]. Female nurses who survived cancer suffer from diminished work performance due to physical restrictions, reduced cognitive functioning due to the adverse effects of cancer treatment, and experience physical and mental fatigue by having to concurrently manage work and treatment [10,13]. Past studies have reported that nurses’ fatigue is related to job stress and turnover intention and influences their QWL. Based on these results, it can be speculated that fatigue among female nurses who survived cancer may contribute to their diminished QWL [8,14,15]. Furthermore, these two very different realities—being a healthcare provider and, at the same time, a cancer survivor—can trigger identity issues, while the prejudice and discrimination against cancer can lead to emotional stress [10,13]. This is because physical and emotional changes, resulting from cancer treatment, can also contribute to a high level of stress, reduced work performance, and difficult interpersonal relationships, which in turn can increase job stress, ultimately decreasing nurses’ satisfaction in the workplace, that is, QWL [16]. Thus, job stress, in addition to fatigue, is speculated to serve as an important predictor of QWL among female nurses who survived cancer.

Studies on the QWL of nurses who have returned to work after having cancer are practically lacking, with only one study that developed the QWL scale for cancer survivors [12] and other studies that examined the level of QWL among cancer survivors and the relationship among fatigue, stress, and workplace spirituality among cancer survivors [16]. As shown here, research on QWL of cancer survivors is still at an inchoate stage, with studies examining QWL among cancer survivors by type of occupation also lacking. In a study on QWL among staff nurses, nurses’ perceived health status [17], educational level and length of career [18], turnover intention [8], job stress [8], and fatigue [15] were identified as the predictors or factors associated with QWL, but no past study has examined QWL among nurses with a chronic disease such as cancer. One survey conducted in Korea reported that healthcare professionals, including nurses, accounted for 30% of all cancer survivors who have returned to work, and this percentage is projected to continue to rise [16,19].

In addition, for nurses, experiencing the process of cancer diagnosis and treatment as a patient deepens their empathy and understanding of patients, which motivates them to provide quality care. Such an experience can also serve as an opportunity to establish a new calling for the nursing profession, beyond simply accepting it as an “occupation” [13]. As such, the concept of self-realization through work can be seen as “workplace spirituality” for nurses [16,20]. Nursing workplace spirituality is important for nurse cancer survivors after return to work, as the sense of accomplishment and self-realization obtained through work can enhance the overall quality of life and the quality of care provided in the care setting [16,20]. Therefore, it is necessary to conduct studies to examine the level of QWL and identify the relevant predictors among female nurse cancer survivors to improve their overall quality of life (QOL) and quality of care.

However, most studies to date have simply set QWL as the dependent variable and used the ordinary least squares (OLS) method for statistical analysis to examine the marginal effects on the mean; for this reason, they have failed to identify the changes in the predictors according to changes in the dependent variable [8,16]. Furthermore, due to human error, it is possible that the influence of a particular variable may be underestimated or overestimated if some traits are focused on a group of people with a low or high distribution of the particular variable [21].

In contrast, quantile regression (QR) enables the examination of the individual influences of each independent variable by quintile. Thus, this technique is useful for identifying causes by examining individual traits in nursing research on humans, who feature a multitude of traits [22]. Considering that individual diversity, the impact of fatigue, job stress, and spirituality will vary among cancer survivors, depending on their QWL, analyses should take this into consideration. However, despite such diversity of traits among individuals, the small number of studies that have examined the characteristics of cancer survivors according to QWL conducted statistical analyses only using the OLS method [16,19].

Thus, with the results of past studies by Wilson and Cleary’s health-related QOL model (1995) as the framework, this study used QR to identify the factors of fatigue, job stress, workplace spirituality, cancer-related characteristics, and job-related characteristics that are specifically associated with different levels of QWL [23].

## 2. Methods

### 2.1. Conceptual Framework

The conceptual framework was established based on Wilson and Cleary’s health-related QOL model (1995) and literature review. According to Wilson and Cleary’s model, physiological factors, symptoms, functional state, and perceived health continuously impact QOL, and symptoms, functional state, perceived health, and QOL are influenced by individual and environmental factors. In the present study, health-related QOL was analyzed as QWL of female nurses who had survived cancer. Fatigue is the most common treatment-related symptom among cancer patients, and it impacts QWL [16,24]. Job stress refers to the physical, psychological, and emotional stress experienced by nurses who are cancer survivors, due to their capabilities of undertaking nursing tasks, and it can be considered an indicator of functional state, showing individuals’ capabilities. Job stress was found to be negatively correlated with job satisfaction as a key component of QWL, and thus is considered a predictor of QWL among cancer survivors [25]. As individual factors we considered age, marital status, number of children, educational level, religion, and cancer-related parameters, and as environmental factors we considered work-related factors, namely, work shift type, job position, and length of career. Further, workplace spirituality, which was identified as a predictor of QWL among cancer survivors, was additionally entered [16].

### 2.2. Study Design

This study employed a cross-sectional survey to identify the factors that affect QWL among registered female nurses who had been diagnosed with cancer.

### 2.3. Sample and Setting

Participants were registered female nurses, who were also cancer survivors, in general hospitals and clinics. The selection criteria were as follows: being a female with a diagnosis of cancer, such as breast cancer, thyroid cancer, and gynecological cancer, and who had returned to work for six months after acute cancer treatment, such as an operation and chemotherapy. The sample size was calculated using the recommendation *n* (sample size): *p* (the number of variables being analyzed) ratio; Gorsuch (1983) argued for minimum ratio of 5 and Everett (1975) recommended that the *n*:*p* ratio should be at least 10. Therefore, a suitable sample size for this study was determined to be 115 [26,27].

### 2.4. Data Collection

We sent an official letter to the nursing unit of five hospitals to request permission for data collection and visited the three hospitals that agreed to it. After providing a detailed explanation of the study’s purpose, participants, data collection procedure, and ethical considerations to the research-related staff, we obtained a final approval from the head of the nursing unit and posted a recruitment poster containing the researcher’s contact information in the nursing unit. Further, we stayed in the nursing unit from 3–4 p.m., the time when the head nurses visit the nursing units, to request them to post the recruitment posters in the nurses’ stations in their units. All participants voluntarily expressed their intention to participate, directly or indirectly, to the researchers. For protecting the privacy of participants, the questionnaires were distributed to the nurses and collected directly by the researchers. In accordance with the ethical process, all participants were given information about the study’s objectives, procedures, and ethical considerations, and then signed an informed consent form. However, due to the low rate of returning to work among nurse cancer survivors in Korea and their reluctance to disclose their cancer history to avoid stigmatization such as prejudice and negative biases within the workplace, there were some difficulties in collecting the data [28]. Thus, we also used snowball sampling to collect data.

### 2.5. Instruments

#### 2.5.1. Fatigue

The Multidimensional Fatigue Scale (MFS) developed for Korean workers by Chang et al. (2005) for general employees was used to measure job fatigue among female nurses who returned to work after surviving cancer [11]. The MFS questionnaire comprised 19 items with a 7-point Likert scale and three subscales: general fatigue (8 items), dysfunction of daily life due to fatigue (6 items), and situational fatigue (5 items). The reliability coefficient of the MFS was 0.93, and 0.95 in our study.

#### 2.5.2. Job Stress

The Expanded Nursing Stress Scale (ENSS) was developed by French et al. (1981) and translated and modified by Kim et al. (2015) to form the Korea version [29]. The questionnaire comprised 48 items with a 4-point Likert scale and nine subscales: death and dying (6 items), conflict with physicians (3 items), inadequate emotional preparation (3 items), problem relating to peers (5 items), problem relating to supervisors (7 items), workload (6 items), uncertainty concerning treatment (7 items), patients and their families (8 items), and discrimination (3 items). The reliability coefficient of the Korean version of the ENSS was 0.95, and 0.95 in our study.

#### 2.5.3. Workplace Spirituality

The Nursing Workplace Spirituality (NWS) scale was developed by Suck and Ko (2016) for Korean nurses [20]. The questionnaire comprises 32 items with a 7-point Likert scale and six sub factors: interaction with job environment (4 items), meaning of nursing (8 items), inner self (6 items), relationship with colleagues (6 items), harmony between workplace and individual (3 items), and transcendence through nursing (5 items). The reliability coefficient of the NWS was estimated as 0.96 in Suk and Ko’s study, and was 0.95 in our study.

#### 2.5.4. Quality of Work Life

The Quality of Nursing Work Life scale was developed by Brooks (2001) and was translated and adapted for Korean clinic nurses [30,31]. The questionnaire comprises 36 items with a 6-point Likert scale and four sub factors: work context (21 items), support system from home/work life (5 items), work design (7 items) and staffing (3 items). The reliability coefficient of the questionnaire was estimated as 0.95 in Kim et al.’s study, and 0.88 in our study.

### 2.6. Statistical Analysis

SPSS statistics version 21 (SPSS, Chicago, IL, USA) and Stata Version 12.0 were used to analyze the survey data. The descriptive statistics were analyzed with SPSS; the reliability coefficients of instruments were estimated by calculating Cronbach’s alpha values. In addition, differences in general characteristics of participants, fatigue, nursing stress, and nursing workplace spirituality according to QWL were analyzed with t-test and ANOVA, and the Pearson correlation among main variables was analyzed. The Durbin–Watson score, the variance inflation factor (VIF), and multiple inner regression for OLS, were applied to investigate the factors affecting the QWL. Finally, Stata version 12.0 was used for QR analysis, to identify such factors according to the level of QWL of cancer survivors among female nurses.

## 3. Results

### 3.1. Differences in Fatigue, Job Stress, Workplace Spirituality, and Quality of Work Life (QWL) according to General Characteristics

The mean age of the participants was 46.8 years (±8.56), with a mean length of career of 22.6 years (±9.67), and mean duration of sick leave due to cancer of 6.48 months (±6.45). The QWL score differed according to age (F = 2.95, *p* = 0.036), education level (F = 3.26, *p* = 0.024), shift work (F = −2.33, *p* = 0.022), job position (F = 5.26, *p* = 0.007), religion (F = −3.20, *p* = 0.002), and type of cancer (F = 5.20, *p* = 0.007). The QWL score was the highest in the 40s group, followed by the 50s group (4.23 ± 0.51), 20s group (3.91 ± 0.72), and 30s group (3.90 ± 0.46). Further, the QWL score was the highest among the master’s group (4.28 ± 0.43), day-shift group (4.22 ± 0.53), nursing manager group (4.35 ± 0.46), and religion group (4.17 ± 0.51). The QWL score was the highest in the gynecologic cancer group (4.64 ± 0.43), followed by the thyroid cancer group (4.17 ± 0.54), and the breast cancer group (4.06 ± 0.53; Table 1)

### 3.2. Score of Fatigue, Job Stress, Workplace Spirituality, and Quality of Work Life (QWL)

Table 2 shows the average scores of the main variables in this study. The average score of QWL was 4.15 ± 0.54 (ranging from 3.11–5.72), the average score of fatigue was 4.52 ± 1.03 (ranging from 1.47–6.79), the average score of job stress was 2.03 ± 0.51 (ranging from 0.65–3.31), and the average of workplace spirituality was 2.88 ± 6.41 (ranging from 2.88–6.41).

### 3.3. Relationships among Affecting Factors and Quality of Work Life (QWL)

Among the main variables, workplace spirituality was significantly associated with QWL, and fatigue was significantly associated with job stress (Table 3).

### 3.4. Comparison between the Ordinary Least Squares (OLS) and Quantile Regression (QR) Results on the Factors Affecting Quality of Work Life (QWL)

To identify the predictors of QWL among female nurses who were cancer survivors, OLS and multiple QR were performed (Table 4). The dependent variable was set to QWL, and the independent variables (explanatory variables) were set to fatigue, job stress, and workplace spirituality, after adjusting for the variables that differed according to QWL, namely, age, education level, type of cancer, shift work, religion, and job position.

In the OLS analysis, breast cancer, shift work, religion, and manager position were the predictors of QWL among general characteristics, and only workplace spirituality was a significant predictor among the key variables. These variables explained 49.7% of the variance. The VIF of the variables included in the OLS were smaller than 3, with a range of 1.0–2.2, confirming the absence of multicollinearity; and the Durbin–Watson d was 1.83, confirming the absence of autocorrelation and thus satisfying the conditions for regression analysis.

In the QR, the predictors of QWL were analyzed by decile units of QWL scores among the participants. The results showed that workplace spirituality was a significant predictor in all deciles, with the exception of the 9th decile of QWL. Fatigue was a predictor in the 2nd, 3rd, and 7th deciles of QWL scores, while job stress was a predictor from the 2nd to 8th decile with the exception of the 5th decile (Table 4).

The degree of the influence of workplace spirituality, job stress, and fatigue, with increasing decile domain of the QWL score, was analyzed. The results showed that the effect size of workplace spirituality was 0.33 (*p* < 0.001) in the 1st decile of the QWL score, which increased to 0.45 (*p* < 0.001) in the 8th decile. The effect size of fatigue decreased from −0.03 to −0.05, with the increasing QWL score from the 2nd decile to 3rd and 7th deciles. The effect size of job stress was 0.07 (*p* < 0.001) in the 2nd decile, which increased to 0.25 (*p* = 0.038) in the 8th decile.

## 4. Discussion

This study investigated the impact of general characteristics, fatigue, job stress, and workplace spirituality on the QWL of female nurse cancer survivors using QR. All participants were women, and the mean age was 46.8 years. QWL was higher among nurses aged 40 years or older, those who worked day shifts, nurse managers, those with religious beliefs, and those who had had gynecologic cancer. With the exception of type of cancer, these results are similar to previous findings, where QWL differed according to age, shift type, job position, and religion [12,16,19]. Regarding cancer type, unlike prior studies, this study limited the type of cancer to three types—breast cancer, thyroid cancer and gynecological cancer—which are the most common types among South Korean women [1].

Cancer survivors experience physical fatigue as a result of cancer treatment, depression, psychological distress (e.g., anxiety), and uncertainty about the future; these contribute to the deterioration of QOL [29]. However, nurse managers who are cancer survivors, who do not work shifts, who are less vulnerable to being exposed to the adverse effects of shift work such as physical fatigue, psychological distress, and poor sleep quality, thus, have higher QWL than staff nurses. Further, spiritual well-being achieved through religious activities can help in maintaining and enhancing QWL [16,20]. However, this study showed that participants’ ages and cancer types were related to differences in the QWL, a finding that is partially supported by previous results showing differences in cancer survivors’ QOL, depending on age and gender [32,33].

There was a positive relationship between workplace spirituality and QWL, but fatigue and stress did not correlate with QWL. Meanwhile, a significant correlation between fatigue and stress was found. This differs from the results of previous studies, in which fatigue, job stress, and workplace spirituality were found to be correlated with QWL [8,16]. These differences can be attributed to variances in participants and instruments, and thus, should be addressed by future studies. The predictors of QWL among this study’s participants were identified as breast cancer, shift work, religion, managerial position, and workplace spirituality, and these variables explained 49.7% of the variance of QWL. Although these results cannot be directly compared with the literature due to a lack of studies on QWL among this population, past findings that workplace spirituality is a predictor of QWL in cancer survivors support our results [16]. Furthermore, our results are partially supported by previous findings that healthy nurses’ workplace spirituality has a positive impact on their QWL [34]. Experiencing cancer is deemed to have a positive impact on QWL, from the perspective that it refreshes nurses’ calling for the nursing profession and improves their empathy toward a patient’s disease and pain, thereby offering an opportunity for self-realization and growth [13]. Therefore, further studies are needed to identify the specific measures that strengthen workplace spirituality among female nurses who survived cancer, to increase their QWL. It is worth mentioning that OLS results did not show fatigue and job stress as significant predictors of QWL, which contradicts previous results where fatigue was significantly correlated with QOL in cancer survivors [35]. The reason for this difference may be due to the fact that the female nurse survivors of cancer in our study had a lower level of fatigue than that of healthy nurses [36], which may be attributable to the stronger perceived need to maintain good health and engage in self-care to recover, resulting from their cancer experience [13].

Regarding job stress, there are differences between the results we obtained and those of a previous study, which found that the job stress of cancer survivors was higher than that of ordinary workers, and job stress was shown to be an influencing factor on the QWL [16]. These differences are believed to stem from the fact that in our study, the mean job stress score among female nurse cancer survivors was lower than that found by another study among general registered nurses without cancer, using the same instrument [8,16]. Furthermore, the difference in these findings can be attributed to the fact that 90% of participants had 11 years or more of work experience and were therefore highly skilled in nursing work. In addition, 37% of participants were nurse managers, who have relatively lower job stress compared to staff nurses [25]. However, our results were partially similar to previous results, where cancer survivor nurses’ job stress did not predict QWL [16,37].

As this study analyzed the overall mean of fatigue and job stress among a single professional group (nurses), the results might not reflect the different characteristics according to the level of QWL. In addition, fatigue is one of the most common physical symptoms experienced by cancer survivors, and the physical and psychological changes cancer survivors have experienced following cancer diagnosis and treatment might impact their QWL by inducing job stress while they readjust to their work lives [13,19]. Therefore, subsequent studies should further examine the features of fatigue and job stress among female nurses who survived cancer and their effects on QWL to re-confirm their impact.

The mean QWL score among our participants was 4.15, which was higher than that among healthy nurses in previous studies [17,30]. This differs from other study findings that cancer survivors have a poorer QOL than non-cancer patients [38]. This can be affected by the lower fatigue and job stress among female nurse cancer survivors, compared to their healthy counterparts, as a previous study observed that fatigue and job stress are significantly correlated with and are important predictors of QWL among cancer survivors [16]. However, nurses’ QWL is heavily influenced by individuals’ subjective emotions and cognition [8], and because the QWL score ranged broadly in our study, from 3.11 to 5.72, there are limitations in examining the relationship between workplace spirituality and QWL using the overall mean of workplace spirituality. Therefore, analyzing the predictors of QWL is essential to devise strategies to improve QWL among this population, with an array of characteristics, and thus it is necessary to identify the predictors of QWL by quantile using QR.

First, workplace spirituality predicted QWL in all deciles with the exception of the 9th decile, which indicated the best QWL among female nurses who survived cancer, confirming that it is a general predictor of QWL, regardless of differences in the level of QWL. Indeed, workplace spirituality seems able to improve QWL for those participants with a low QWL, and satisfies the psychological and mental needs to maintain QWL among those with high QWL, by granting personal joy and meaning through the practice of nursing activities and helping achieve self-realization by contributing to nursing performance and organizational goals [30]. Thus, it is important to explore and implement specific interventions for workplace spirituality to promote self-satisfaction and self-realization through occupational calling, as this would contribute to improving QWL and, ultimately, to improving overall QOL by maintaining and managing QWL.

Fatigue predicted QWL in the low QWL groups (2nd and 3rd deciles) and in the relatively high QWL group (7th decile), and the effect size was similar across the deciles. This result is similar to that of a previous study, where fatigue was identified as a predictor of QOL in the low QOL groups (10th, 25th percentiles) among gastric cancer survivors [39]. As with healthy nurses, fatigue can be an important factor not only in one’s QWL, which indicates subjective satisfaction, but also in work, such as work competence, efficiency, and patient safety, in nurse cancer survivors who returned to work [14]. Thus, QWL should be improved by reducing fatigue, and maintained and managed by evaluating and ameliorating individuals’ health status, workload, and form of work.

Job stress was a predictor of QWL in the low QWL groups (2nd and 3rd deciles), moderate QWL group (4th decile), and high QWL groups (6th–8th deciles), suggesting that job stress has an overall impact on QWL among nurses who returned to work after cancer. An interesting result was that the effect size of job stress was 0.07 in the 2nd decile but increased to 0.25 in the 8th decile, which indicates that the degree of impact of job stress increases with increasing QWL. Job stress occurs when workers’ abilities and resources needed to carry out a task do not fall in line with the demands [40]. The resulting job stress can serve as a motivator for self-improvement, which can improve work abilities and performance, thereby improving QWL. As shown here, the QR confirmed that the major variables of fatigue, job stress, and workplace spirituality have varying effects depending on the level of QWL among the participants. Thus, it is necessary to examine the effects and characteristics of variables according to the level of QWL among this population, and to explore specialized strategies and measures based on the results.

In this study, workplace spirituality, breast cancer, thyroid cancer, shift work, religion, and managerial position were identified as the predictors of QWL in the lowest QWL group (1st decile). The effect size of workplace spirituality was 0.33, which was lower than that in the 2nd–8th deciles (0.42–0.45). This suggests that, while workplace spirituality is an important predictor for people with both high and low QWL, the influence of other general characteristics should be taken into consideration for those in the 1st decile of QWL. In particular, the effect size of shift work was 0.38 in the 1st decile of QWL, and this was higher than that in the next decile. This suggests that, in addition to the internal and emotional support necessary to strengthen workplace spirituality, considerations for types of shifts in light of the health statuses are needed for nurses returning to work after cancer, to improve the QWL among those with low QWL. This indicates that, to improve QWL for these nurses, it is necessary to provide support for health management at the organizational level, and to improve the work environment, to allow them to manage the various stresses that can occur during nursing work. In addition, we surmise that enhancing workplace spirituality will increase the value and meaning of nursing work, helping female nurses who survived cancer to experience a greater sense of achievement and satisfaction in their nursing work, which could, in turn, increase their QWL [8,13]. As such, it will be important to explore integrative measures to aid control of fatigue and job stress, and to increase workplace spirituality; these measures should be applied to the management and improvement of QWL of nurses who survived cancer. This is because, as with healthy nurses, fatigue, job stress, and workplace spirituality are statistically significantly correlated [41] and workplace spirituality in cancer survivors impacts the regulation of job stress and job satisfaction, a similar concept to QWL [19].

In addition, fatigue, job stress, and workplace spirituality predicted QWL also in a high QWL group (7th decile), while job stress and workplace spirituality predicted QWL in the 8th decile. This highlights the need for management of fatigue and job stress and strengthening of workplace spirituality also for participants with high QWL. However, the effect sizes of fatigue and workplace spirituality in the 7th decile were similar to those in the 2nd and 3rd deciles, while the effect size of job stress increased from 0.07 in the 2nd decile to 0.25 in the 8th decile, highlighting the importance of job stress management for those with high QWL. According to our results, job stress triggered by motivation to achieve organizational goals and take up new challenges for change seems to have a positive influence on QWL [40]. However, excessive job stress has an adverse impact on job satisfaction, QWL, and organizational commitment, and consequently increases turnover intention among healthy nurses [8]. Thus, it hinders job retention and successful return to work by female nurses after cancer treatment, who are exposed to physical symptoms such as fatigue and psychological and social health problems [13]. For this reason, subsequent studies should further examine the impact of job stress on QWL among these individuals. Further, in addition to examining the features of individuals’ job stress, measures to manage job stress according to the level of QWL should be explored to manage job stress among female cancer survivors. As shown here, the type of cancer, shift work, religion, job position, and workplace spirituality, which were identified as predictors of QWL in female nurses who survived cancer in the OLS analysis, had varying levels of effects depending on the quantile of QWL scores in the QR analysis. Furthermore, fatigue and job stress, which did not predict QWL in the OLS analysis, did predict QWL depending on the amount of change of QWL in the QR analysis. Thus, further replication studies are needed to examine the impact of fatigue and job stress on QWL.

The OLS method estimates predictors based on the mean QWL scores, meaning it neglects the diversity of the participants. However, the QR technique, which is used to identify predictors in accordance with the amount of change in the dependent variable, has rarely been used in nursing research [42]. The QR technique will be useful in nursing research, as it enables estimation of each explanatory variable according to the amount of change of the variable. One limitation of this study is that only a subset of female nurse cancer survivors was analyzed, which limits the generalizability of the findings. To address this, subsequent studies should use a larger sample.

## 5. Conclusions

The difference between the factors influencing the QWL of female nurse cancer survivors was determined by the QR method. Unlike the OLS method, which is commonly used in the study of influencing factors, the QR method has the advantage of being able to identify the characteristics of the participants according to their QWL, and to propose customized arbitration measures for this purpose. Based on the results of the current study, we can present the following important points. First, workplace spirituality was a strong influence factor of QWL in all groups, with the exception of the 9th decile, in the QR analysis. This finding demonstrates the influence of workplace spirituality on the QWL of cancer survivor nurses and highlights the importance of exploring ways to improve spirituality in the workplace. Second, unlike in the OLS analysis method, where only workplace spirituality was identified as an influencing factor among the main independent variables, in the QR analysis, fatigue and job stress appeared as influencing factors in the 2nd and 3rd deciles for the low QWL group, and in the 7th decile for the high QWL group. Therefore, it can be proposed that job stress and fatigue control strategies based on the level of QWL among cancer survivor nurses who have returned to work. Furthermore, organizations should analyze and provide the necessary support to improve nurses’ QWL by adopting measures considerate of their health status, such as shorter work shifts. In addition, organizations can establish practical employee health systems to improve the quality of work life of female nurse cancer survivors. Examples include breaks during working duty, especially during the night shift, and time and economic supports for cancer treatment.

## Figures and Tables

**Table 1:** Differences in fatigue, job stress, workplace spirituality, and quality of work life (QWL) according to general characteristics (*n* = 115).

				Fatigue		Job Stress		Workplace Spirituality		Quality of Work Life	
		*n*	(%)	Mean ± SD							
Age	20s	4	(3.5)	5.00	±1.20	2.22	±0.70	4.09	±0.52	3.91	±0.72
	30s	26	(22.6)	4.96	±0.71	2.17	±0.46	4.57	±0.72	3.90	±0.46
	40s	39	(33.9)	4.43	±0.88	2.02	±0.46	5.29	±0.54	4.25	±0.57
	50s	46	(40.0)	4.29	±1.20	1.94	±0.55	5.05	±0.66	4.23	±0.51
	F(*p*)			2.81	(0.043)	1.29	(0.282)	9.38	(<0.001)	2.95	(0.036) *
Marital status	single	24	(21.0)	4.79	(±1.04)	2.11	(±0.44)	4.90	(±0.62)	4.27	(±0.55)
	married	91	(79.0)	4.44	(±1.03)	2.00	(±0.53)	4.99	(±0.73)	4.11	(±0.54)
	t(*p*)			1.41 (0.161)		0.84 (0.399)		−0.54 (0.592)		1.24 (0.217)	
Education	college	10	(8.7)	4.37	(±0.66)	1.87	(±0.47)	4.79	(±1.01)	3.87	(±0.57)
	university	40	(34.8)	4.68	(±1.10)	2.07	(±0.44)	4.84	(±0.74)	4.16	(±0.61)
	over Master	65	(56.5)	4.44	(±1.03)	2.04	(±0.53)	5.11	(±0.61)	4.08	(±0.46)
	F(*p*)			0.837 (0.436)		0.631 (0.534)		2.32 (0.105)		1.49 (0.231)	
Work type	shift work	33	(29.0)	4.44	(±1.18)	1.93	(±0.44)	4.90	(±0.73)	3.97	(±0.55)
	full-time	82	(71.0)	4.55	(±0.96)	2.07	(±0.53)	5.02	(±0.69)	4.22	(±0.53)
	F(*p)*			−0.53 (0.597)		−1.32 (0.190)		−0.82 (0.412)		−2.33 (0.022)	
Position	nurses	72	(63.0)	4.57	(±0.97)	2.07	(±0.51)	4.89	(±0.74)	4.03	(±0.56)
	manager	43	(37.0)	4.42	(±1.10)	1.96	(±0.51)	5.16	(±0.61)	4.35	(±0.46)
	T(*p*)			0.40 (0.669)		1.02 (0.365)		2.77 (0.067)		5.26 (0.007)	
Religion	Yes	91	(80.0)	4.47	(±1.05)	2.02	(±0.51)	5.14	(±0.60)	4.17	(±0.51)
	No	24	(20.0)	4.72	(±0.92)	2.06	(±0.48)	4.38	(±0.73)	4.06	(±0.64)
	T(*p)*			0.79 (0.430)		1.11 (0.268)		−2.02 (0.046)		−3.20 (0.002)	
Cancer type	Breast ca ^a^	66	(57.0)	4.45	(±1.05)	2.06	(±0.46)	4.98	(±0.66)	4.06	(±0.53)
	Thyroid ca ^b^	39	(34.0)	4.72	(±1.02)	2.05	(±0.55)	4.86	(±0.76)	4.17	(±0.54)
	gynecologic ca ^c^	10	(9.0)	4.18	(±0.71)	1.71	(±0.55)	5.51	(±0.48)	4.64	(±0.43)
	F(*p*) Scheffé			1.45 (0.239)		2.17 (0.119)		3.47 (0.034)		5.20 (0.007)	
										a, b < c **	
Cancer stage	I	77	(67.0)	4.65	(±1.01)	2.05	(±0.53)	4.96	(±0.71)	4.17	(±0.54)
	II	29	(25.0)	4.10	(±1.03)	1.84	(±0.42)	5.01	(±0.76)	4.05	(±0.48)
	Over III	9	(8.0)	4.68	(±0.75)	2.45	(±0.26)	5.14	(±0.46)	4.26	(±0.76)
	F(*p*)			3.25 (0.042)		5.60 (0.005)		0.30 (0.740)		0.75 (0.476)	
Work experience (year)	<5	4	(3.5)	4.78	(±0.53)	2.13	(±0.13)	5.05	(±0.62)	4.34	(±0.38)
	5–10	7	(6.0)	4.75	(±1.02)	2.09	(±0.58)	4.17	(±0.77)	3.81	(±0.64)
	11–15	24	(21.0)	4.84	(±0.96)	2.12	(±0.44)	4.62	(±0.67)	3.97	(±0.47)
	16–20	7	(6.0)	4.98	(±0.63)	2.13	(±0.43)	4.84	(±0.52)	4.07	(±0.37)
	>20	73	(63.5)	4.33	(±1.06)	1.97	(±0.54)	5.20	(±0.63)	4.24	(±0.56)
	F(*p*)			1.77 (0.141)		0.53 (0.712)		6.90 (<0.001)		2.03 (0.095)	
Illness period (year)	<1	63	(54.8)	4.53	(±1.09)	2.08	(±0.51)	4.93	(±0.74)	4.20	(±0.50)
	1–5	8	(6.9)	4.91	(±1.08)	2.05	(±0.47)	5.19	(±0.52)	4.25	(±0.52)
	6–10	33	(28.7)	4.57	(±0.79)	1.90	(±0.48)	5.00	(±0.68)	4.11	(±0.64)
	>10	11	(9.6)	3.95	(±1.13)	2.09	(±0.61)	5.11	(±0.71)	3.95	(±0.53)
	F(*p*)			0.81 (0.490)		1.58 (0.199)		0.97 (0.411)		0.45 (0.717)	

* There is no difference among age groups by Scheffé test. ** As a Post-hoc after-ANOVA test, represents the difference in QWL level among cancer types, a: mean of QWL of breast cancer group, b: mean of QWL of Thyroid cancer group, c: mean of QWL of Gynecologic cancer group. Gynecologic cancer group’s QWL level is higher than other groups.

**Table 2:** Scores of fatigues, job stress, workplace spirituality, and quality of work life (*n* = 115).

	Fatigue	Job Stress	WS *	QWL
**Fatigue**	1.47	6.79	4.52	1.03
**Job stress**	0.65	3.31	2.03	0.51
**WS**	2.88	6.41	4.99	0.70
**QWL**	3.11	5.72	4.15	0.54

* Workplace Spirituality.

**Table 3:** Relationships among affecting factors and quality of work life (QWL; *n* = 115).

	QWL	Fatigue	Job Stress	WS
**QWL**	1			
**Fatigue**	−0.040	1		
**Job stress**	0.600	0.339 **	1	
**WS**	0.559 **	−0.063	−0.004	1

** *p* < 0.01.

**Table 4:** Affecting factors according to quality of work life (QWL) levels among nurse cancer survivors by quantile regression (*n* = 115).

Variable	OLS	QR								
		Q0.1	Q0.2	Q0.3	Q0.4	Q0.5	Q0.6	Q0.7	Q0.8	Q0.9
	B (SE)	Coef. (SE)								
Age	0.01	0.01	0.01 ***	0.01	0.01 *	0.01	0.01 *	0.01	0.01	0.01
	(0.01)	−(0.01)	(0.00)	(0.00)	(0.00)	(0.00)	(0.00)	(0.00)	−(0.01)	−(0.03)
Education	0.01	−0.05	0.06 ***	0.01	0.01	−0.06	−0.05	−0.11 ***	−0.06	−0.14
	(0.05)	−(0.04)	−(0.01)	−(0.01)	−(0.03)	−(0.03)	−(0.04)	−(0.02)	−(0.06)	(0.21)
Breast cancer	0.52 ***	−0.60 ***	−0.53 ***	−0.54 ***	−0.46 ***	−0.33 ***	−0.40 ***	−0.40 ***	−0.38 *	−0.34
	(0.15)	−(0.12)	−(0.04)	−(0.03)	−(0.06)	−(0.08)	−(0.10)	−(0.05)	−(0.15)	−(1.06)
Thyroid cancer	0.15	−0.54 ***	−0.30 ***	−0.29 ***	−0.32 ***	−0.19 *	−0.28 *	−0.32 ***	−0.27	−0.09
	(0.10)	−(0.12)	−(0.04)	−(0.03)	−(0.08)	−(0.09)	−(0.11)	−(0.06)	−(0.17)	−(1.21)
Shift work	0.22 *	0.38 **	−0.00	−0.00	0.02	0.11	0.18 *	0.12 **	0.36 **	0.19
	(0.10)	−(0.13)	−(0.02)	−(0.02)	−(0.05)	−(0.06)	−(0.07)	−(0.04)	−(0.12)	−(0.46)
Religion	0.38 **	0.39 **	0.50 ***	0.28 ***	0.40 ***	0.18 *	0.25 **	0.29 ***	0.51 **	0.61
	(0.13)	−(0.13)	−(0.03)	−(0.02)	−(0.07)	−(0.07)	−(0.09)	−(0.06)	−(0.16)	−(0.60)
Position	0.26 *	0.34 **	0.32 ***	0.34 **	0.32 ***	0.25 ***	0.21 **	0.27 ***	0.09	0.23
	(0.10)	−(0.12)	−(0.02)	−(0.02)	−(0.05)	−(0.06)	−(0.08)	−(0.04)	−(0.11)	−(0.55)
Fatigue	−0.01	−0.08	−0.03 ***	−0.05 ***	0.03	0.03	0.03	0.05 *	0.04	−0.02
	(0.04)	−(0.04)	−(0.01)	−(0.01)	−(0.02)	−(0.03)	−(0.03)	−(0.02)	−(0.05)	−(0.32)
Job stress	0.14	0.05	0.07 ***	0.09 ***	0.09 *	0.08	0.25 ***	0.22 ***	0.25 *	0.34
	(0.08)	−(0.07)	−(0.02)	−(0.01)	−(0.04)	−(0.05)	−(0.06)	−(0.04)	−(0.12)	−(0.65)
Workplace spirituality	0.43 ***	0.33 ***	0.42 ***	0.46 ***	0.46 ***	0.44 ***	0.42 ***	0.45 ***	0.45 ***	0.52
	(0.06)	−(0.05)	−(0.02)	−(0.01)	−(0.03)	−(0.04)	−(0.05)	−(0.03)	−(0.07)	−(0.31)
R^2^	0.49	0.35	0.32	0.33	0.34	0.34	0.36	0.36	0.36	0.32

OLS = Ordinary Least Square; QR = Quantile Regression; Coef. = Coefficient, SE = Standard Errors; * *p* < 0.05; ** *p* < 0.01, *** *p* < 0.001.
